# Linking Electronic Relaxation Dynamics and Ionic Photofragmentation Patterns for the Deprotonated UV Filter Benzophenone-4

**DOI:** 10.1021/acs.jpclett.1c00423

**Published:** 2021-03-15

**Authors:** Natalie G. K. Wong, Conor D. Rankine, Caroline E. H. Dessent

**Affiliations:** † Department of Chemistry, University of York, Heslington, York, YO10 5DD, U.K.; ‡ School of Natural and Environmental Sciences, Newcastle University, Newcastle-upon-Tyne, NE1 7RU, U.K.

## Abstract

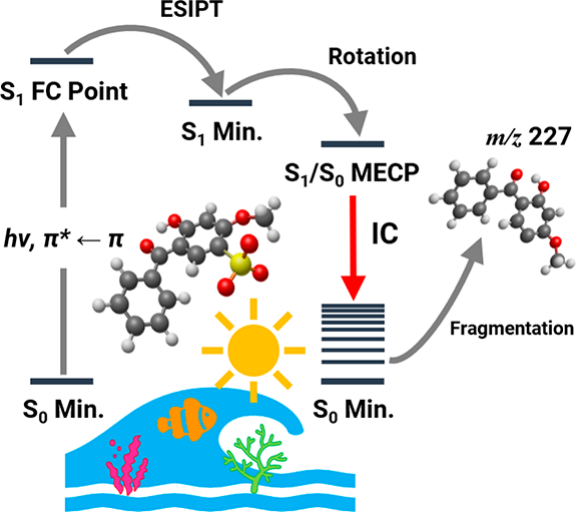

Understanding how deprotonation impacts the photophysics of UV filters is critical to better characterize how they behave in key alkaline environments including surface waters and coral reefs. Using anion photodissociation spectroscopy, we have measured the intrinsic absorption electronic spectroscopy (400–214 nm) and numerous accompanying ionic photofragmentation pathways of the benzophenone-4 anion ([BP4–H]^−^). Relative ion yield plots reveal the locations of the bright S_1_ and S_3_ excited states. For the first time for an ionic UV filter, *ab initio* potential energy surfaces are presented to provide new insight into how the photofragment identity maps the relaxation pathways. These calculations reveal that [BP4–H]^−^ undergoes excited-state decay consistent with a statistical fragmentation process where the anion breaks down on the ground state after nonradiative relaxation. The broader relevance of the results in providing a basis for interpreting the relaxation dynamics of a wide range of gas-phase ionic systems is discussed.

Laser spectroscopy has been increasingly applied over recent years to characterize the intrinsic photophysics of UV filters to provide a more robust understanding of molecular-level sunscreen action.^1^ Both solution and gas-phase experiments have been performed, and while the solution phase can constitute an environment closer to that of a commercial sunscreen mixture,^1−5^ gas-phase studies are of particular value in providing data that can readily be interpreted by high-level theory.^5−9^ While several neutral sunscreens have been the subject of gas-phase investigations, protonated and deprotonated analogues have been studied much more sparsely.^5^ These experiments are important given that a number of aquatic environments are alkaline (e.g., surface water and coral reefs),^10,11^ so that the understanding of how deprotonation affects photostability has important environmental implications.

Very recently, laser-interfaced mass spectrometry (LIMS) has been used to probe the photophysics of several ionic sunscreen systems in detail.^12−16^ These studies reveal that protonation and deprotonation can dramatically affect the sunscreen’s UV absorption profile. Information on decay dynamics (and hence the intrinsic sunscreen efficiency), however, has only been inferred indirectly in these experiments, through attempting to match the photofragmentation products against the corresponding thermal fragmentation products to elucidate whether excited-state decay is statistical or nonstatistical.^17,18^ This is a general problem for gaseous studies of ionic systems that extends well beyond the specific field of sunscreens,^17,19−23^ since there are currently few experiments where direct time-resolved measurement of ionic photofragments is possible.^24,25^

Here, we present the first laser spectroscopy study of benzophenone-4, BP4 (Scheme 1), in its deprotonated form. BP4 is structurally similar to oxybenzone (OB; Scheme 1), which is one of the most widely investigated sunscreens, both experimentally and theoretically.^5,7,26−29^ Studies have revealed that the sunscreen action of OB arises from excited-state intramolecular proton transfer (ESIPT) yielding the *keto* form of neutral oxybenzone, which then undergoes ultrafast internal conversion (IC) from the excited- to ground-state potential energy surface and efficiently thermalizes the excess energy.^27−29^ Notably, for both deprotonated and protonated OB, the observed photofragmentation patterns were interpreted as indicative of nonstatistical excited-state decay, due to disruption of the *keto–enol* moiety.^15^ BP4 provides an important analogue to study in this respect, since it contains a strongly acidic sulfonic acid group in addition to the OB *keto–enol* site. Deprotonation of BP4 will therefore produce the sulfonate monoanion, leaving the crucial *keto–enol* site intact for uninterrupted operation of the ultrafast nonradiative relaxation mechanism. Our aim here is to compare the photofragmentation behavior of deprotonated OB and BP4 to investigate whether excited-state decay is in fact nonstatistical and statistical, respectively. For the first time for a deprotonated UV filter, we apply quantum chemical calculations to obtain *ab initio* potential energy surfaces and hence gain direct physical insight into how the photofragment identity maps the nonradiative relaxation channels.

**Scheme 1:**
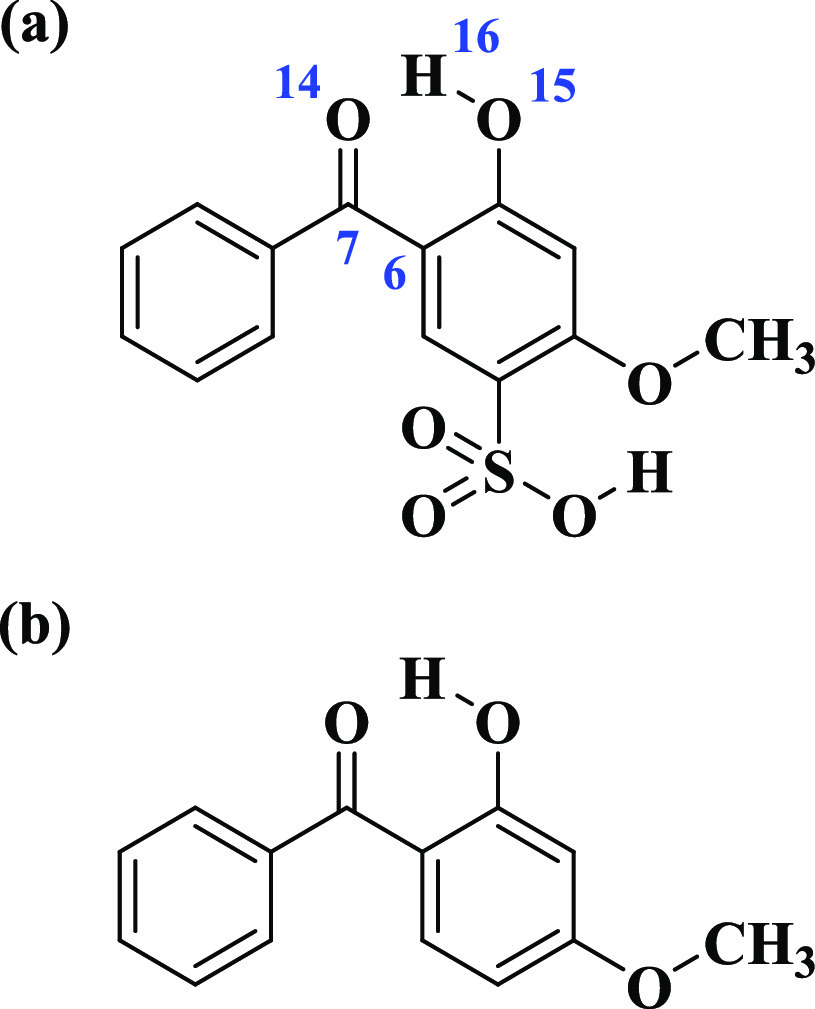
Molecular Structures of (a) Benzophenone-4 (BP4) and (b) Oxybenzone (OB)

LIMS action spectroscopy was used to record gaseous ion photodepletion and photofragment spectra of [BP4–H]^−^ (Section S1).^12−16^ The photodepletion spectrum can be considered to be equivalent to the gaseous absorption spectrum in the limit where radiative decay is absent. Figure 1a displays the photodepletion spectrum of mass-selected [BP4–H]^−^ (*m*/*z* 307) over the range 3.1–5.8 eV (400–214 nm), displaying strong absorption across the UV. To aid in the discussion of the photofragment production spectra, the key spectral features are labeled **I**–**IV**, with bands **I** and **II** being the two distinct UVA and UVB absorption bands, peaking at 3.5 and 4.1 eV, respectively. Band **III** increases gradually in intensity in the UVC between 4.5 and 5.0 eV, and leads into band **IV** which is a strong, broad feature (onset *ca*. 5.0 eV) that extends further into the UVC.

**Figure 1:**
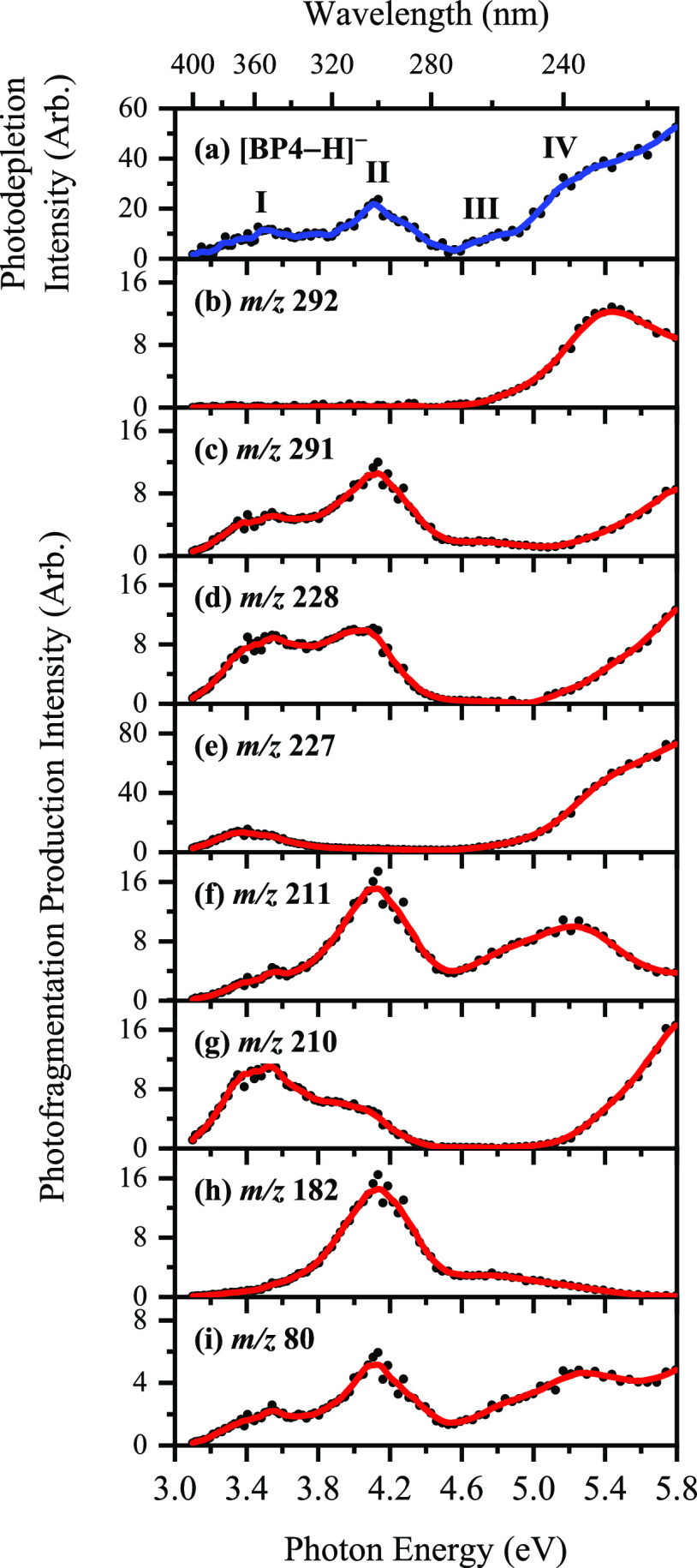
(a) Gas-phase absorption (photodepletion) spectrum of [BP4–H]^−^ (*m*/*z* 307). (b–i) Photofragment production spectra of the eight major photofragments of [BP4–H]^−^: *m*/*z* 292, 291, 228, 227, 211, 210, 182, and 80. The solid line is a five-point adjacent average of the data points.

We next turn to the photofragment ions produced following photoabsorption by [BP4–H]^−^. Photofragmentation is extensive, with over 20 photofragments being observed. Figure 1b–i display the action spectra of the most prominent photofragments, with minor photofragments being reported in Section S3. The most intense photofragment ion is observed at *m*/*z* 227 (eq 1d), corresponding to loss of neutral SO_3_ via a heterolytic cleavage mechanism of the C–S bond of the parent anion. *m*/*z* 227 is produced with high intensity across the UVA and lower-energy UVC regions. The other major photodissociation channels of [BP4–H]^−^ are given in eqs 1a–1h, with the fragmentation channels discussed further in Section S6. We note that free radical formation is dominant.
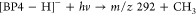
1a
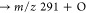
1b
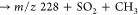
1c
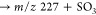
1d
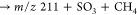
1e
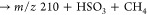
1f
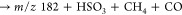
1g
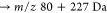
1h

In Figure 1b–i, several distinctive spectral profiles are evident for the various photofragment ions. All the photofragment action spectra, except for those of *m*/*z* 292 and 182 fragments, show a prominent peak in the UVA (ca. 3.5 eV), corresponding to the photodepletion feature **I**. A subsequent band, peaking at 4.1 eV, is also evident for the *m*/*z* 291, 228, 211, 182, and 80 fragments, in the region of feature **II**. The growth in production of several of the photofragment ions (*m*/*z* 291, 228, 227, 210, and 80) beyond 5.0 eV traces the profile of feature **IV** (Figure 1a). We note that the vertical detachment energy (VDE) for [BP4–H]^−^ is calculated as 5.19 eV, so that most of the spectral range lies below the electron detachment threshold. It is interesting to note that, for the *m*/*z* 292 photofragment, production peaks at 5.4 eV, possibly indicating that a dipole-bound excited state is accessed in this region that decays with formation of *m*/*z* 292.^30,31^Section S4 discusses electron detachment further.

Figure 2a presents the relative photofragment ion yields of [BP4–H]^−^ as a function of photoexcitation energy, highlighting several maxima that can be attributed to photoexcitation into different electronic states. It is evident that, in both the UVA and low UVC regions, the relative ion yield of the *m*/*z* 227 photofragment is ca. 50% larger than other photoproduct ions. Conversely, within the range 3.8–5.0 eV, the production of fragment ions *m*/*z* 211 and 182 (and to a lesser extent *m*/*z* 291) increases significantly in comparison to the remaining ionic photofragments.

**Figure 2:**
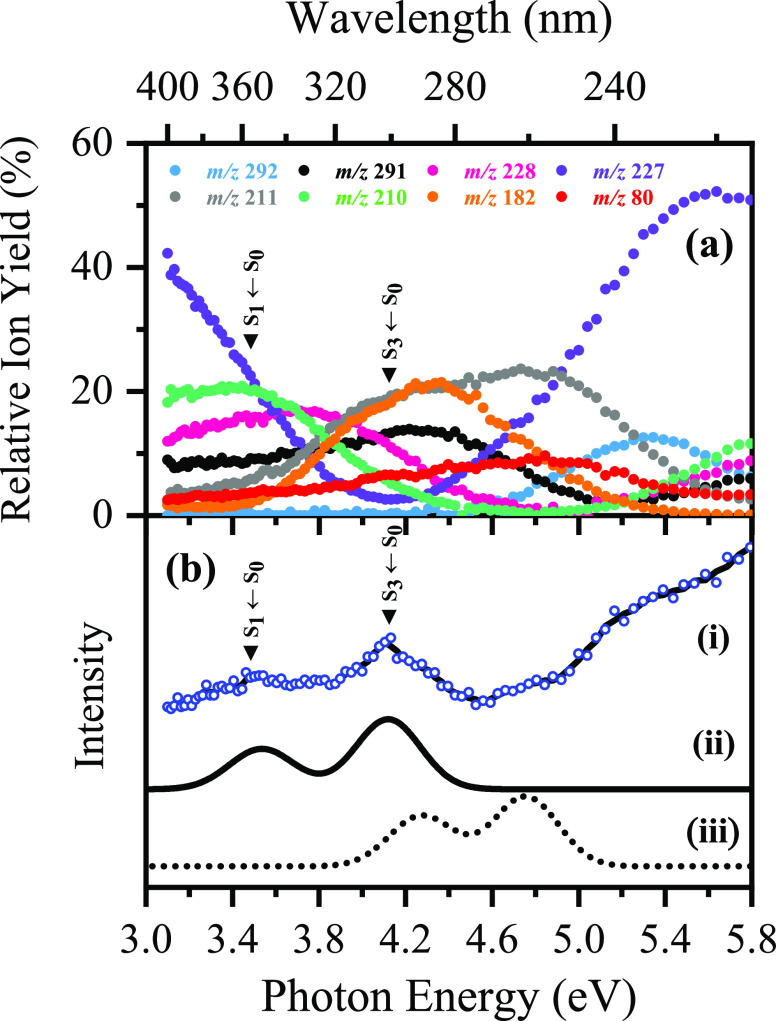
(a) Relative ion yield plot highlighting the eight most intense photofragments of [BP4–H]^−^ seen upon laser excitation between 3.1 and 5.8 eV. (b) Gas-phase experimental photodepletion spectrum (i) vs theoretical UV absorption spectra calculated at the (ii) ADC(2)/MP2/ma-*def*2-SV(P) and (iii) ωB97X-D/ma-*def*2-SV(P) levels. The optically bright S_1_ ← S_0_ and S_3_ ← S_0_ ππ* transitions are indicated.

To probe the thermal fragmentation pathways of [BP4–H]^−^ on its electronic ground state, higher-energy collisional dissociation (HCD) was employed (Table 1, Figure 3, and Section S5).^14,21^ These measurements are essential to identify which ions are secondary products, formed when a precursor species fragments at high internal energy.^32^ They are also important, as any photofragments not observed in HCD can be identified as purely photochemical products. At relatively low collisional energies (20–42% HCD), the most intense fragment ion is *m*/*z* 227, with *m*/*z* 291, 228, and 210 also being produced in significant quantities. This indicates that thermal breakdown of the electronic ground state of [BP4–H]^−^ is associated with the molecule fragmenting along a number of different pathways. Production of the *m*/*z* 227, 228, and 291 ions all decreases at higher energies, concomitant with the *m*/*z* 211 fragment increasing. (We note that the *m*/*z* 291 fragment persists to higher collisional energies than *m*/*z* 227 and 228, indicating higher relative stability.) The HCD results therefore reveal that *m*/*z* 211 is a secondary fragment from *m*/*z* 227, 228, and 291 at higher internal energy. Similarly, *m*/*z* 210 appears to decrease as the *m*/*z* 182 ion increases.

**Table 1:** Summary of the Ionic Fragments of Deprotonated BP4 (*m/z* 307) Produced upon UV Laser Photoexcitation and Higher-Energy Collisional Dissociation (HCD) at 40% and 70% HCD Energies (Proposed Structures Are Outlined in Table S1)

	Observed in HCD^b^		
Ionic mass fragment (*m*/*z*)^a^	40%	70%	Observed in UV laser photoexcitation^b^
292	√ (xw)^c^	–	√ (m)
291	√ (m)	√ (w)	√ (m)
228	√ (m)	√ (vw)	√ (m)
227	√ (s)	√ (vw)	√ (vs)
211	√ (w)	√ (vs)	√ (m)
210	√ (m)	√ (vw)	√ (m)
182	√ (m)	√ (m)	√ (m)
80	√ (w)	√ (m)	√ (w)

a Determined with mass accuracy >0.3 amu.

b Very strong (vs), strong (s), moderate (m), weak (w), very weak (vw), and extremely weak (xw).

c HCD fragment *m*/*z* 292 is observed to peak at 34% HCD energy, with a relative ion intensity of <2%.

**Figure 3:**
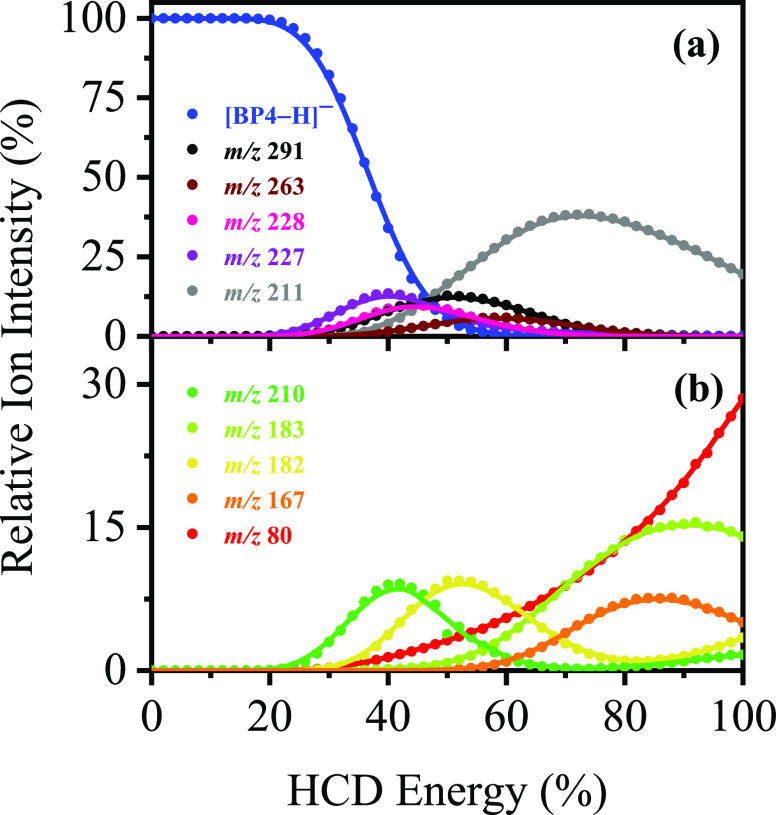
Parent ion dissociation curves for [BP4–H]^−^ highlighting its ten most intense thermal fragments between 0% and 100% HCD energy. The curved lines are a five-point adjacent average of such data points and are provided as a viewing guide, to emphasize the profile for each individual fragment.

Since the *m*/*z* 227, 228, 291, and 211 fragments dominate both the UV photofragmentation of [BP4–H]^−^ and thermal (HCD) fragmentation, photofragmentation of the anion can be categorized as predominantly statistical (ergodic) over the spectral range studied.^17,18^Section S5 discusses the more minor HCD fragments and branching between the minor fragmentation pathways in more detail.

To explore whether this picture of statistical photofragmentation for [BP4–H]^−^ is credible, quantum chemical calculations were performed to characterize the excited-state potential energy surfaces (Section S1). The *C*_1_-symmetric S_0_ minimum-energy geometry of [BP4–H]^−^ was located at the ωB97X-D level (Table S2), with key excited-state parameters (ωB97X-D and ADC(2) levels) summarized in Table 2.

**Table 2:** Summary of Vertical Excitation Energies, Δ*E*, Oscillator Strengths, *f*, and Characters of the S*_n_* ← S_0_ (*n* = 1, 2, 3) States As Evaluated at the ωB97X-D/ma-*def*2-SV(P) and ADC(2)/MP2/ma-*def*2-SV(P) Levels

		ωB97X-D		ADC(2)	
State	Char.	Δ*E* (eV)	*f*	Δ*E* (eV)	*f*
S_1_	ππ*	4.272	0.256	3.533	0.156
S_2_	*n*π*	4.357	0.010	3.701	0.004
S_3_	ππ*	4.756	0.365	4.120	0.273

Figure 2b displays the calculated UV absorption spectra of [BP4–H]^−^, along with the experimental photodepletion spectrum. We assign the two bands observed in the UVA/UVB regions of the experimental [BP4–H]^−^ photodepletion spectrum (**I** and **II**) as the optically bright S_1_ ← S_0_ and S_3_ ← S_0_ ππ* transitions, respectively. The excellent agreement between the calculated spectra at both the ωB97X-D and ADC(2) levels and the experimental spectrum (Figure 2b) is notable, both in terms of state identities, relative peak positions, and intensities. At the ADC(2) level, quantitative agreement with experiment is obtained ‘*out of the box*’, whereas, at the ωB97X-D level, the vertical excitation energies of the S*_n_* ← S_0_ (*n* = 1, 2, 3) states are characteristically overestimated (ca. 0.7 eV) but in good qualitative agreement.

Based on our understanding of the sister molecule, OB,^15,29^ [BP4–H]^−^ can be expected to relax on the S_1_ state via ESIPT. A *C*_1_-symmetric S_1_ minimum-energy geometry for [BP4–H]^−^ was located ca. 4.5 Å Da^–1/2^ from the Franck–Condon point (Table S3). The S_1_ minimum-energy geometry is accessed via ESIPT from the Franck–Condon point, with the H_16_ atom bound to O_1__5_ migrating across to O_1__4_. ESIPT follows a direct excited-state relaxation coordinate and is consequently expected to occur promptly postphotoexcitation to the S_1_ state. Post-ESIPT, [BP4–H]^−^ can access the S_1_/S_0_ crossing seam at an S_1_/S_0_ minimum-energy crossing point (MECP). An S_1_/S_0_ MECP was located ca. 18.2 and 18.0 Å Da^–1/2^ from the Franck–Condon point and S_1_ minimum-energy geometry, respectively (Table S4). The S_1_/S_0_ MECP is accessed via torsion of C_6_–C_7_ and is characterized by the aromatic rings being rotated into a near-perpendicular conformation, effectively closing the gap between the S_0_ and S_1_ states.

To map the S_0_ ← S_1_ IC channel, potential energy surfaces have been constructed between the key geometries via linear interpolation of internal coordinates (LIIC). Independent single-point energy calculations have been carried out at each one of 25 interpolated geometries, respectively, with the calculated potential energy surfaces presented in Figure 4a.

**Figure 4:**
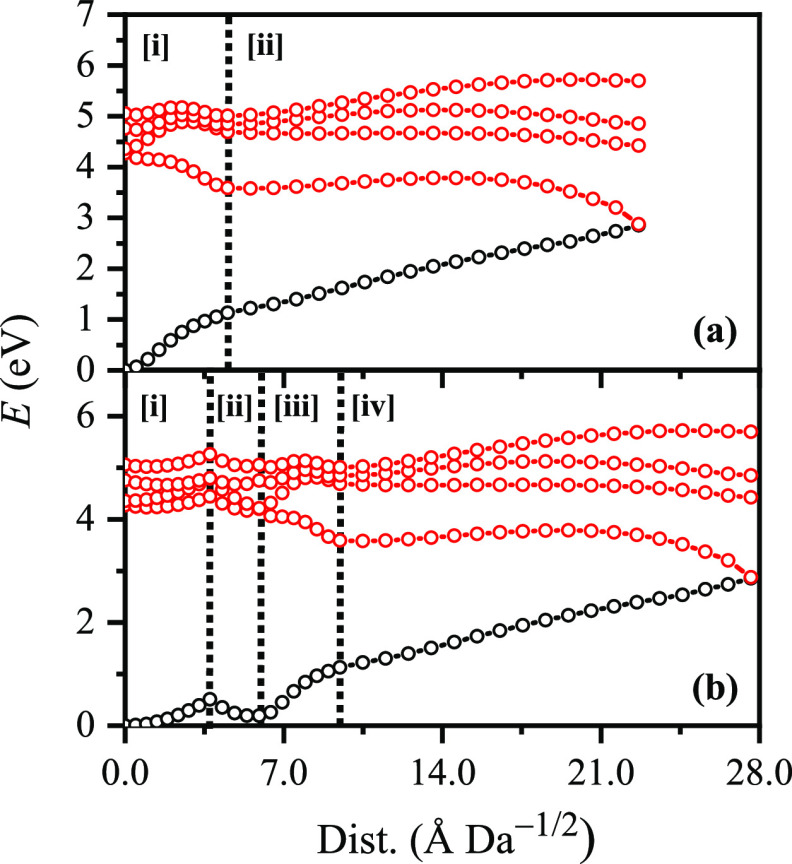
(a) Energies of the S_0_ state (black) and excited singlet states (red) between (i) the S_0_ and S_1_ minimum-energy geometries and (ii) the S_1_ minimum-energy geometry and the S_1_/S_0_ MECP. (b) Energies of the S_0_ state (black) and excited singlet states (red) between (i) the S_0_ minimum-energy geometry and the S_3_/S_2_ MECP, (ii) the S_3_/S_2_ MECP and the S_2_/S_1_ MECP, (iii) the S_2_/S_1_ MECP and the S_1_ minimum-energy geometry, and (iv) the S_1_ minimum-energy geometry and the S_1_/S_0_ MECP. Points were generated via linear interpolation of internal coordinates (LIIC). Energies were evaluated at the ωB97X-D/ma-*def*2-SV(P) level.

The picture to emerge here is similar to that described for OB by Karsili et al.,^29^ which is consistent with experiments which identified a subpicosecond lifetime for the IC channel.^27^ The quantum-chemical calculations reported here are not able to give information on the time scale that the S_1_/S_0_ crossing seam is accessed (although they could be readily coupled to excited-state dynamics simulations such as nonadiabatic mixed-quantum-classical or trajectory surface-hopping dynamics, to directly obtain this information). However, given the similar potential energy surface morphologies of [BP4–H]^−^ and OB around the key *keto–enol* region, it is reasonable to expect that it is ultrafast (i.e., subpicosecond) and, therefore, able to outcompete other processes efficiently, e.g., excited-state fragmentation, radiative decay, and intersystem crossing. (For ISC; T_1_ ← S_1_ and T_2_ ← S_1_ spin–orbit couplings are on the order of ca. 5–10 cm^–1^ along the LIIC channel: Section S8.) The calculations are therefore entirely consistent with our deduction from the experimental results of nonradiative relaxation followed by statistical fragmentation on the hot ground state. This leads to ejection of SO_3_ as the initial dominant channel, as the C–S bond is the weakest bond in [BP4–H]^−^.^33^ Loss of SO_3_ is commensurate with production of the *m*/*z* 227 fragment, both from excitation at feature **I**, i.e., the lowest-energy optically bright state, and, crucially, from the HCD production curves (Figure 3).

For the feature **II** region, which corresponds to excitation into the optically bright S_3_ state, the calculations predict decay pathways that appear similar to those outlined for feature **I**. Figure 4b shows the S_3_/S_2_ and S_2_/S_1_ MECPs that have been located (Tables S5–S6), showing that both lie close to (ca. 3.7 and 2.1 Å Da^–1/2^, respectively), and downhill of, the respective Franck–Condon point. Thus, S_3_ excitation is predicted to lead to a prompt S_1_ ← S_2_ ← S_3_ cascade of population. After arriving on the S_1_ state close to the Franck–Condon point, ESIPT and ultrafast S_0_ ← S_1_ IC will proceed as described above. We speculate that S_0_ ← S_1_ IC, when the S_1_ state is accessed indirectly (from above; i.e., postphotoexcitation into the S_3_ state) as opposed to directly (postphotoexcitation to the S_1_ state), could be even more efficient, since accessing the S_3_/S_2_ MECP and the S_2_/S_1_ MECP directly accesses the proton transfer and torsional coordinates, respectively, that are necessary to subsequently access the S_1_/S_0_ crossing seam. This could be tested in future work by either excited-state dynamics simulations^34^ and/or time-resolved experiments.^24,25^

The differences in fragment production on excitation at features **I** and **II** can then be explained as follows. Excitation at feature **I** (the S_1_ state) leads to fission of the C–S bond after nonradiative relaxation (as previously observed for UVB filter 2-phenylbenzimidazole-5-sulfonic acid),^14^ producing primarily the *m*/*z* 227, 228, and 291 fragments. Excitation at feature **II** (the S_3_ state) will also lead to fission of the C–S bond after nonradiative relaxation and the production of the *m*/*z* 227, 228, and 291 fragments. However, as a greater amount of photon energy is pumped into the system (4.1 eV versus 3.5 eV), these fragments possess enough internal energy to undergo secondary fragmentation. The reduction in photofragment intensity can be seen first for *m*/*z* 227, then for *m*/*z* 228, and finally for *m*/*z* 291, exactly mirroring the measured relative stability of these ions from the HCD measurements (Figure 3). (We note that similar arguments can be applied to the *m*/*z* 210 and 182 photofragments, where comparison to the HCD data reveals that the *m*/*z* 182 ion persists to higher internal energy.) All of these photofragments therefore produce the *m*/*z* 211 fragment as a secondary product: indeed, the *m*/*z* 211 fragment dominates the medium-high HCD energy range between 42% and 80% HCD energies.

In summary, we have reported the gaseous UV absorption spectrum and photofragmentation profile of [BP4–H]^−^ acquired via LIMS. For the first time for an ionic UV filter, *ab initio* potential energy surfaces are presented to provide new insight into the relaxation pathways. The calculations predict that, in the regions of both the optically bright S_1_ ← S_0_ and S_3_ ← S_0_ ππ* transitions, excited state relaxation will occur via nonradiative decay, associated with a statistical excited state decay process. In the photodissociation experiments, the observed photofragments mirror those observed upon thermal breakdown of the electronic ground state. Importantly, the photon-energy dependent production spectra of the numerous photofragments mirror the fragment production curves in the HCD collisional activation measurements. This is clear evidence of statistical decay, driven by fragmentation on a hot ground state surface, which in turn demonstrates that deprotonated BP4 is behaving like an efficient UV filter. However, the results presented here are of broader importance, as they provide a theoretical basis to support the widely adopted argument linking ionic photofragmentation patterns and decay dynamics that has been used for interpreting the behavior of key gaseous ionic systems including nucleobases and nucleotides.^17,19−23,35,36^

## Supporting Information Available

Experimental and computational methodology; photodepletion laser power dependence measurements; additional photofragment action spectra; electron detachment yield versus photodepletion yield interpretation; higher-energy collisional dissociation (HCD) production spectra; further discussion of deprotonated benzophenone-4 fragmentation channels; optimized Cartesian coordinate tables; further computational results; schematic structure of deprotonated benzophenone-4 (PDF)
