# Supplementary material for: Linking Electronic Relaxation Dynamics and Ionic Photofragmentation Patterns for the Deprotonated UV Filter Benzophenone-4
Source: J Phys Chem Lett. 2021 Mar 15;12(11):2831–6. doi: 10.1021/acs.jpclett.1c00423 (PMC8041369; doi:10.1021/acs.jpclett.1c00423)
Supplement: Supplementary file 1 — jz1c00423_si_001.pdf [file jz1c00423_si_001.pdf]

Supporting Information

Linking Electronic Relaxation Dynamics and Ionic Photofragmentation Patterns for the Deprotonated UV Filter Benzophenone-4

Natalie G. K. Wong,[a] Conor D. Rankine,[b] and Caroline E. H. Dessent[a*]

[a] Department of Chemistry, University of York, Heslington, York, YO10 5DD, UK.

[b] School of Natural and Environmental Sciences, Newcastle University, Newcastle-upon-Tyne, NE1 7RU, UK.

* Corresponding author: Email: caroline.dessent@york.ac.uk

Supporting Information:

S1. Experimental and computational methodology

S2. Photodepletion laser power dependence measurements

S3. Additional photofragment action spectra

S4. Electron detachment yield versus photodepletion yield interpretation

S5. Higher-energy collisional dissociation (HCD) production spectra

S6. Further discussion of deprotonated benzophenone-4 fragmentation channels

S7. Optimized Cartesian coordinate tables

S8. Further computational results

S9. Schematic structure of deprotonated benzophenone-4

S1. Experimental and computational methodology

Gas-phase UV photodissociation experiments were conducted in an AmaZon SL electrospray ionization quadrupole ion-trap (ESI-QIT) mass spectrometer (Bruker Daltonics Inc., Billerica, MA, USA), which was modified to allow for laser-interfaced mass spectrometry (LIMS). This instrument has the advantages of a commercial mass spectrometer, coupled with the ability to record UV-visible photodissociation spectra in a routine manner.

Benzophenone-4 (2-hydroxy-4-methoxybenzophenone-5-sulfonic acid; sulisobenzone; BP4) was purchased from Sigma Aldrich, Inc. (St. Louis, MO, USA). Solutions of BP4 (~10^{-4} M) in HPLC-grade acetonitrile (Fisher Scientific, Inc., Pittsburgh, PA, USA) were introduced into the mass spectrometer by ESI using typical instrumental parameters in the negative ion mode: nebulizing gas pressure: 14.0 psi; injection rate: 0.33 mL/hr; drying gas flow rate: 10.0 L/min; and capillary temperature: 160 °C.

Deprotonated BP4 ($[BP4–H]^-$) was mass selected and isolated in the ion trap prior to laser irradiation. Photons were produced by a 10 Hz Nd:YAG (Surelite™, Amplitude Laser Group, San Jose, CA, USA) pumped OPO (Horizon™, Amplitude Laser Group) laser, giving ~0.3 mJ across the range 400-214 nm (3.1-5.8 eV). A laser step size of 2 nm was used and the laser beam was focused as has been described previously.[1–4] Photofragmentation experiments were conducted with an ion accumulation time of 2 ms and a fragmentation time of 100 ms, thereby ensuring that each ion packet interacted with one laser pulse to minimize the likelihood of multiphoton events. A laser power dependence study for $[BP4–H]^-$ is outlined in Section S2. As fluorescence is also negligible here,[1–3,5,6] the UV-excited gaseous ion will fragment upon excited state relaxation, yielding an action absorption spectrum by photodepletion (see Figures 1b-i and Section S3). Photodepletion was measured as a function of the scanned wavelength, with photofragment production recorded simultaneously (see Eqs. S1-3):

$$\text{Photodepletion intensity} = \frac{\ln\left(\frac{\text{Int}_{\text{OFF}}}{\text{Int}_{\text{ON}}}\right)}{\lambda \times \text{P}} \qquad \text{(Eq. S1)}$$

$$\text{Photofragmentation intensity} = \frac{\left(\frac{\text{Int}_{\text{FRAG}}}{\text{Int}_{\text{OFF}}}\right)}{\lambda \times \text{P}} \qquad \text{(Eq. S2)}$$

$$\text{Relative ion yield} = \text{Int}_{\text{FRAG}}/\text{Int}_{\text{PFT}} \qquad \text{(Eq. S3)}$$

where Int_{OFF} and Int_{ON} are the peak parent ion intensities with laser off and on, respectively; Int_{FRAG} is the fragment intensity with the laser on; λ is the excitation wavelength (nm); P is the tunable laser pulse energy (mJ); and Int_{PFT} is the sum of the photofragment ion intensities with the laser on.

Higher-energy collisional dissociation (HCD) was performed on $[\text{BP4–H}]^-$ using an Orbitrap™ Fusion Tribid mass spectrometer (Thermo Fisher Scientific, Waltham, MA, USA) with an ESI source, run in the negative ion mode between 0-100% collisional energy.[2,7,8] This technique provides tandem mass spectrometry and was operated at a flow rate of 3.0 µL/min, with the following parameters: spray voltage: -2500 V; sheath gas flow rate: 10; aux. gas flow rate: 2.0, ion transfer tube temperature: 290 °C; vaporizer temperature: 20 °C; MS^2 detector: ion trap; scan rate: enhanced; MS^2 AGC target: 10,000; MS^2 max. injection time: 100 ms; and RF lens: 60%.

All density functional theory (DFT) calculations were carried out using ORCA (v4.2.1).[9,10] All second-order algebraic diagrammatic construction/Moller-Plesset perturbation theory [ADC(2)/MP2] calculations were carried out using TURBOMOLE (v6.3.1).[11]

DFT and time-dependent DFT (TDDFT) calculations employed the *ω*B97X-D density functional of Head-Gordon *et al.*[12] and used the resolution-of-identity (RI) approximation for Coulomb and Hartree-Fock exchange integrals (RI-JK). ADC(2)/MP2 calculations employed the CC2 routines implemented in TURBOMOLE,[13–16] and used the frozen-core approximation; the 25 lowest-energy core orbitals were frozen in all ADC(2)/MP2 calculations. A tightened SCF convergence criterion

of 1.0×10^{-8} a.u. was used in all calculations; tightened convergence criteria of 1.0×10^{-6} and 3.0×10^{-5} a.u. were used for the energy change and RMS gradient, respectively, in all geometry optimizations. The proper convergence of all geometry optimizations to real minima was verified *via* vibrational frequency inspection. Minimum-energy crossing points (MECP) between electronic states were located *via* a home-built external optimizer following the approach of Martinez *et al.*[17]

The minimally-augmented (*ma*-)*def*2-SV(P) basis set of Truhlar *et al.*[18] was used throughout; where required for RI-(TD)DFT, a density-fitting auxiliary basis set was generated following the approach of Neese *et al.*[19]

S2. Photodepletion laser power dependence measurements

Laser power measurements were conducted on $[BP4–H]^-$ at four absorption maxima: 3.5 eV (354 nm), 4.1 eV (300 nm), 5.3 eV (234 nm), and 5.4 eV (230 nm). The plot displayed in Figure S1 shows that of the parent ion photodepletion intensities (Int_{OFF} – Int_{ON}) at such photon energies. Following standard protocol, such data has been plotted and fit to a power function.[1,2,20] The resultant slope is thereby proportional to the number of absorbed photons implicated in the experiment. Multiphoton events *via* instantaneous absorption of multiple photons in the Franck-Condon region are negligible as the laser beam is only softly focused through the ion-trap region. The slopes at all four photon energies measure at less than 1, confirming that photodepletion of $[BP4–H]^-$ at 0.3 mJ is evidently not multiphoton in nature.

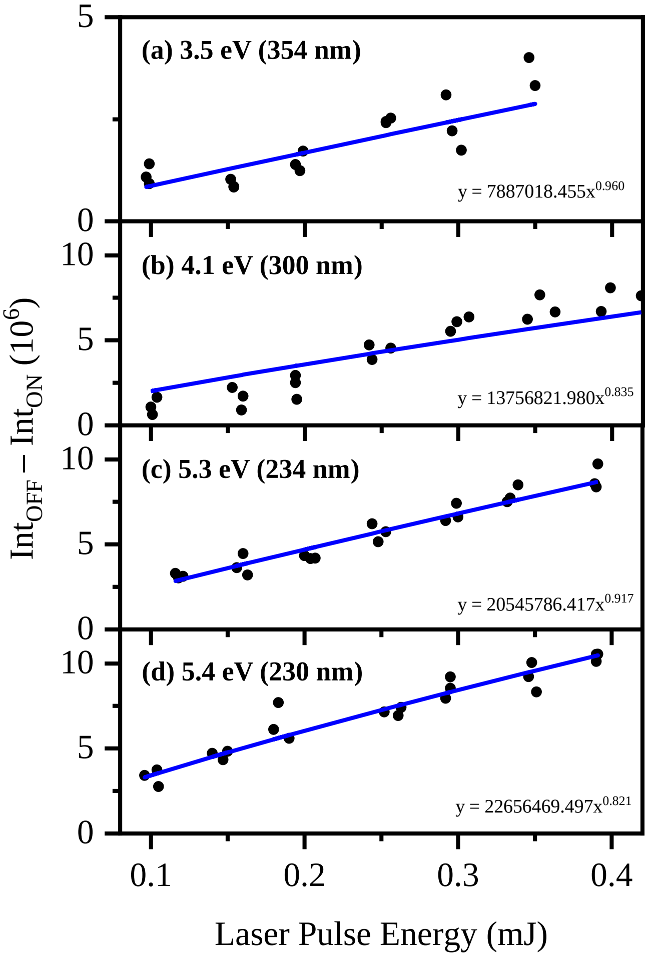

Figure S1. Power dependence measurements for $[BP4–H]^{-}$ at four absorption maxima of **(a)** 3.5 eV (354 nm), **(b)** 4.1 eV (300 nm), **(c)** 5.3 eV (234 nm), and **(d)** 5.4 eV (230 nm).

S3. Additional photofragment action spectra

It is apparent that the UV photofragmentation of $[BP4–H]^-$ is extensive. To provide a comprehensive assessment of the majority of the photofragments observed, Figures S2-3 show the photofragment action spectra of the next 11 most intense photofragments, all deemed relatively minor in comparison to those presented in the main text (Figures 1b-i).

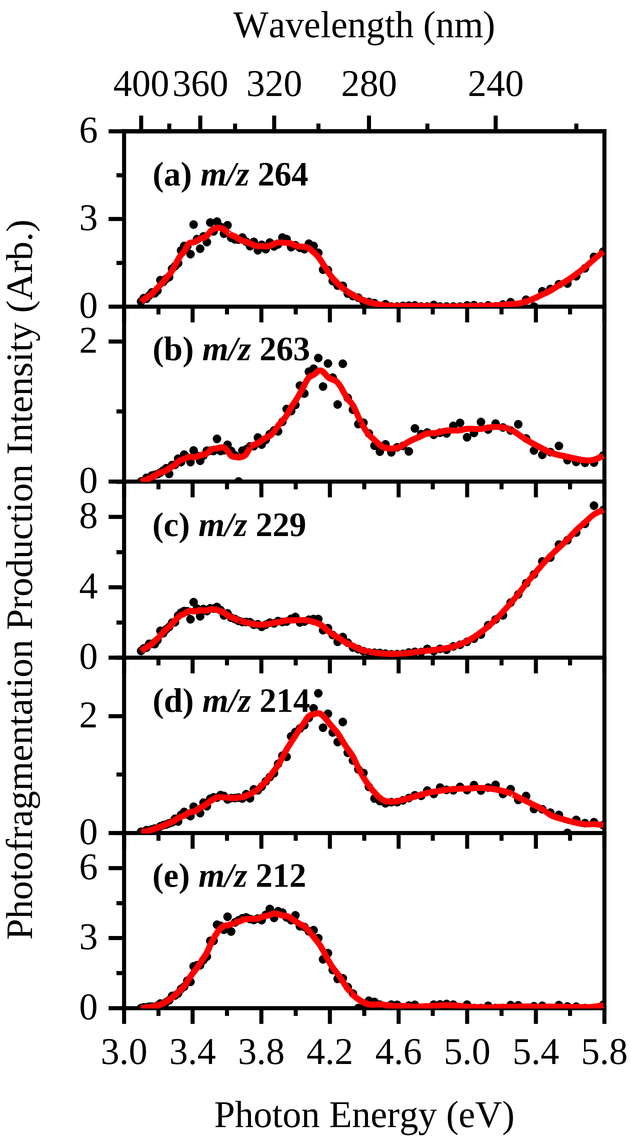

Figure S2. Additional photofragment action spectra for the first set of minor $[BP4–H]^-$ fragments observed at *m/z* 264, 263, 229, 214, and 212. The solid line is a five-point adjacent average of the data points.

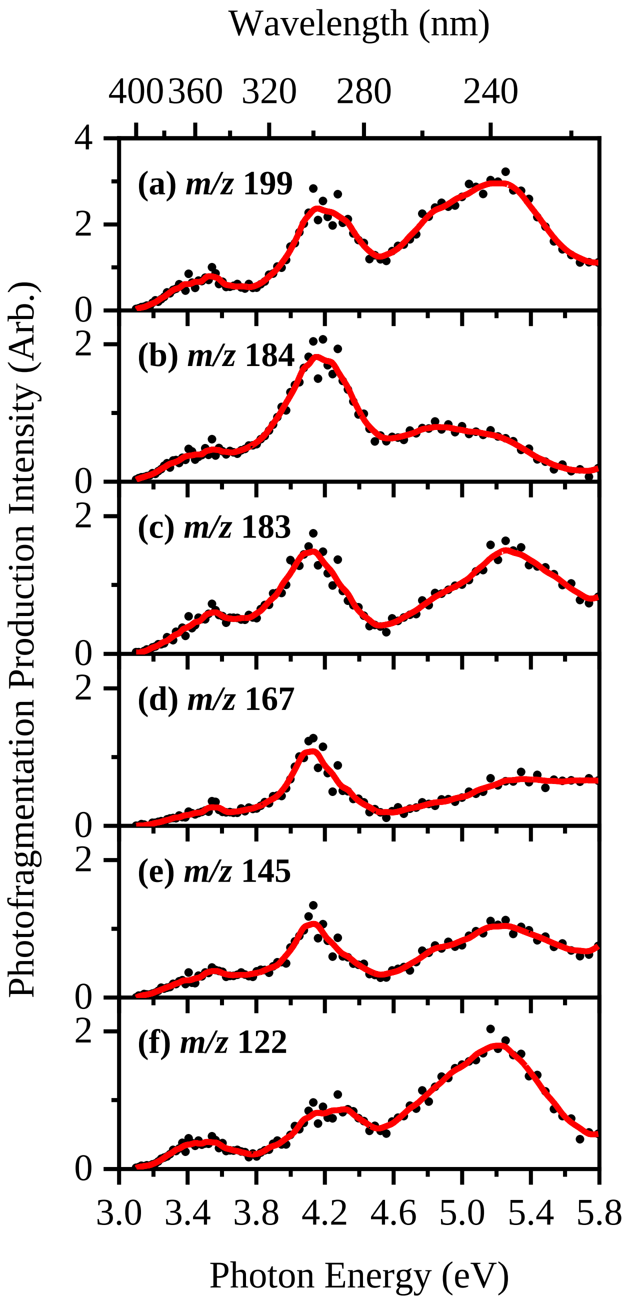

Figure S3. Additional photofragment action spectra for the second set of minor $[BP4–H]^{-}$ fragments observed at *m/z* 199, 184, 183, 167, 145, and 122. The solid line is a five-point adjacent average of the data points.

S4. Electron detachment yield versus photodepletion yield interpretation

Electron loss has been regarded as the most dominant photofragmentation channel for gaseous sunscreen anions previously studied within our group, *e.g.* 2-phenylbenzimidazole-5-sulfonic acid (PBSA) and oxybenzone (OB), and this has been in line with their calculated vertical detachment energies (VDEs).[1,2] The VDE of $[BP4–H]^{-}$ (5.19 eV) indicates, however, that the electrons here are being detached below this. Nevertheless, the decay of $[BP4–H]^{-}$ through electron detachment is less concerning, given that photodetachment is quenched on solvation for anionic molecules, *e.g.,* in sunscreen formulations.

Electron loss is not directly measurable within our instrument and thus can only be calculated *via* the use of Eqs. S4-5, assuming that any photodepletion ions that are not detected as ionic photofragments are instead losing an electron. Note that fragment ions with *m/z* < 50 are not detectable in our mass spectrometer since low masses fall outside the mass window of the ion trap.

Electron detachment yield (ED*) spectra were calculated by assuming that any depleted ions not detected as ionic photofragments are decaying *via* means of electron detachment, as determined using Eq. S4. This analysis assumes that both the parent ions and photofragments are detected equally in the mass spectrometer. In Figure S4 where we present ED* spectra, we overlay such data with the photodepletion yield (PD*) for ease of comparison; PD* is the normalized photodepletion ion count (Eq. S5), which provides the most straight-forward comparison to the ED* (Eq. S4):

$$ED^* = \frac{\frac{(Int_{OFF} - Int_{ON}) - Int_{PFT}}{Int_{OFF}}}{\lambda \times P} \qquad \text{(Eq. S4)}$$

$$PD^* = \frac{\frac{Int_{OFF} - Int_{ON}}{Int_{OFF}}}{\lambda \times P} \qquad \text{(Eq. S5)}$$

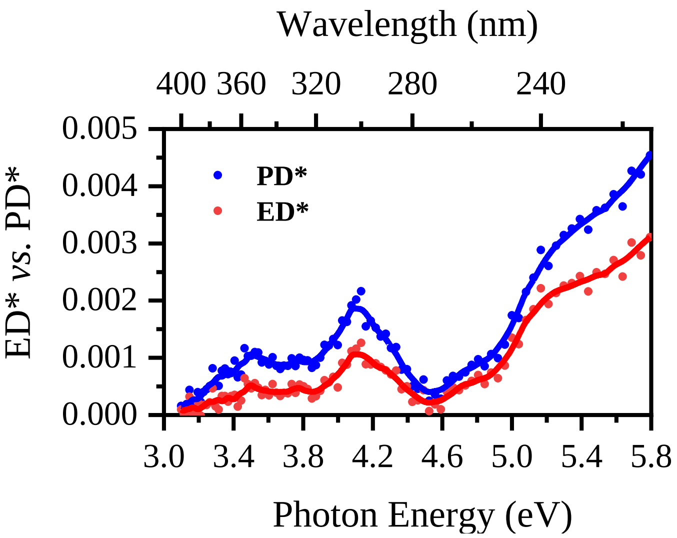

Figure S4. Electron detachment yield (ED*; red) *vs.* photodepletion yield (PD*; blue) of [BP4–H]$^-$. The solid line is a five-point adjacent average of the data points.

Such yields do not significantly overlap as greatly within the UVA and UVB regions in comparison to previously studied gas-phase iodide ion-nucleobase clusters,[21] but does strongly suggest that [BP4–H]$^-$ decays predominantly through means of electron detachment. Similar effects have also been observed in the case of the adenosine monophosphate anions.[7]

S5. Higher-energy collisional dissociation production spectra

Figures 3 and S5 display the major and minor HCD fragmentation curves for [BP4–H]$^-$, respectively, illustrating a clear onset of thermal fragment production at 20% HCD energy. Hereafter, a great number of thermal fragment ions can be observed. The identifiable thermal fragment ions are summarized in Table 1, and their postulated structures presented in Section S6.

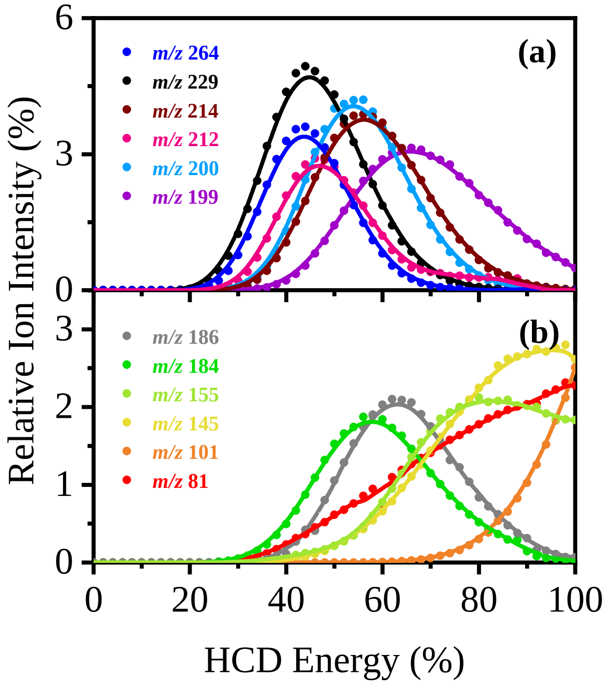

Figure S5. Photofragment action spectra for the minor [BP4–H]$^-$ HCD fragments observed at **(a)** *m/z* 264, 229, 214, 212, 200, and 199, and at **(b)** *m/z* 186, 184, 155, 145, 101, and 81. The solid lines are a five-point adjacent average of the data points.

S6. Further discussion of deprotonated benzophenone-4 fragmentation channels

The main text presents and discusses the implications of the major photofragments of $[BP4–H]^-$ observed for *m/z* 292, 291, 228, 227, 211, 210, 182, and 80 in Figures 1b-i of the main text.

The major dissociation channels of $[BP4–H]^-$ are again given in Eqs. 1a-h and their proposed structural assignments outlined in Table S1. A condensed version is available in the main text (Table 1).

Table S1. Proposed structures for the major ionic fragments of $[BP4–H]^-$ (*m/z* 307) produced upon UV laser photoexcitation and higher-energy collisional dissociation (HCD) at 40% and 70% HCD energies.

Ionic mass fragment (*m/z*)[a]	Proposed structure of fragment	Accompanying neutral fragment lost	Observed in HCD[b]		Observed in UV laser photoexcitation[b]
			40%	70%	
**292**	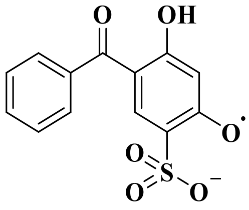	CH_3	✓ (xw)[c]	-	✓ (m)
**291**	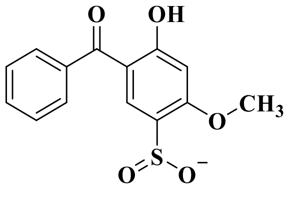	O	✓ (m)	✓ (w)	✓ (m)
**228**	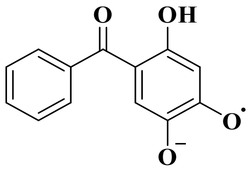	$SO_2 + CH_3$	✓ (m)	✓ (vw)	✓ (m)

Ionic mass fragment (*m/z*)[a]	Proposed structure of fragment	Accompanying neutral fragment lost	Observed in HCD[b]				Observed in UV laser photoexcitation[b]	
			40%		70%			
**227**	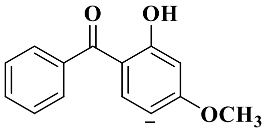	SO_3	✓	(s)	✓	(vw)	✓	(vs)
**211**	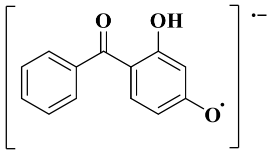	$SO_3 + CH_4$	✓	(w)	✓	(vs)	✓	(m)
**210**	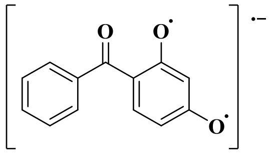	$HSO_3 + CH_4$	✓	(m)	✓	(vw)	✓	(m)
**182**	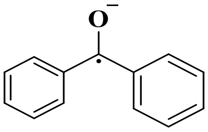	$HSO_3 + CH_4 + CO$	✓	(m)	✓	(m)	✓	(m)
**80**	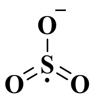	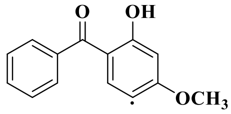	✓	(w)	✓	(m)	✓	(w)

[a] Determined with mass accuracy > 0.3 amu. [b] Very strong (vs), strong (s), moderate (m), weak (w), very weak (vw), and extremely weak (xw). [c] HCD fragment *m/z* 292 is observed to peak at 34% HCD energy, with a relative ion intensity of <2%.

The loss of 15 Da from $[BP4–H]^-$ is consistent with the loss of a methyl group to form the phenoxy radical ion at *m/z* 292 (Eq. 1a), an observation in line with previous work mapping degradation of anisole[22] and deprotonated OB.[1] The assignment of fragments *m/z* 291 (Eq. 1b) and *m/z* 228 (Eq. 1c) (Table S1) was informed using results from the dissociation of benzenesulfonic acid and benzenesulfinic acid.[23–25] This leads us to assign the *m/z* 291 fragment as loss of an oxygen atom from the SO_3^- moiety of $[BP4–H]^-$,[23] with the *m/z* 228 fragment arising from SO_2 ejection from a

rearranged Ph–O–SO_2^-.[23–25] Finally, the observed *m/z* 80 fragment ion can be assigned as a radical SO_3^-, arising from homolytic cleavage of the C–S bond.[23]

S7. Optimized Cartesian coordinate tables

Table S2. Optimized Cartesian coordinates in Å and ground-state SCF energies, E_{SCF}, in atomic units (a.u.) for the S_0 minimum-energy geometry of $[\mathrm{BP4{-}H}]^-$ at the *ω*B97X-D/ma-*def*2-SV(P) level.

E_{SCF} = -1388.57549345			
C	-0.221823	-2.474803	-0.169963
C	-1.603388	-2.356399	-0.219380
C	-2.259796	-1.123178	-0.067627
C	-1.484431	0.055007	0.140452
C	-0.108941	-0.087281	0.191964
C	0.571665	-1.307683	0.017039
C	2.025734	-1.417411	0.021824
C	2.899645	-0.195327	-0.020550
C	2.630324	0.898969	-0.851174
C	3.504442	1.984836	-0.884751
C	4.648867	1.988961	-0.088816
C	4.929039	0.895032	0.732993
C	4.064432	-0.195648	0.757314
O	2.587897	-2.521916	0.052048
O	0.327076	-3.686993	-0.304755
H	1.297719	-3.570681	-0.177224
O	-3.587198	-1.247939	-0.152467
C	-4.551797	-0.261584	0.198804
H	-4.562619	0.554397	-0.535452
H	-4.339858	0.165119	1.188310
H	-5.510208	-0.797412	0.204407
S	-2.077745	1.780564	0.268760
O	-0.833317	2.577668	0.277872
O	-2.840145	1.849913	1.535092
O	-2.900647	1.970580	-0.947346
H	-2.211392	-3.250037	-0.365031
H	0.449074	0.831265	0.379763
H	1.736785	0.908418	-1.478744
H	3.277387	2.836745	-1.529973
H	5.325067	2.848219	-0.107212
H	5.826649	0.892962	1.357408
H	4.277982	-1.065224	1.383514

Table S3. Optimized Cartesian coordinates in Å and ground-state SCF energies, E_{SCF}, in atomic units (a.u.) for the S_1 minimum-energy geometry of $[BP4–H]^-$ at the ωB97X-D/ma-*def*2-SV(P) level.

E_{SCF} = -1388.53218793			
C	-0.189887	-2.426716	-0.233872
C	-1.606019	-2.331474	-0.281191
C	-2.324692	-1.113947	-0.165787
C	-1.599187	0.064941	0.008142
C	-0.174595	-0.048337	0.168476
C	0.555785	-1.188063	-0.071235
C	2.003609	-1.274848	0.103742
C	2.927667	-0.173048	0.037203
C	2.617097	1.065700	-0.580650
C	3.548424	2.098697	-0.603340
C	4.815817	1.933596	-0.038266
C	5.147897	0.705490	0.550083
C	4.228189	-0.331991	0.585643
O	2.492968	-2.446416	0.477174
O	0.412608	-3.544054	-0.316546
H	1.777166	-3.133830	0.234764
O	-3.651000	-1.277169	-0.268088
C	-4.582317	-0.388119	0.332754
H	-4.783715	0.467847	-0.324290
H	-4.200977	-0.009801	1.292572
H	-5.493557	-0.979943	0.494090
S	-2.201953	1.778049	-0.070194
O	-0.963458	2.541880	-0.332798
O	-2.798417	2.043326	1.256794
O	-3.168326	1.780022	-1.185847
H	-2.172039	-3.249083	-0.451719
H	0.323851	0.872478	0.479737
H	1.654761	1.219162	-1.070791
H	3.277453	3.043470	-1.081528
H	5.542335	2.750217	-0.061557
H	6.139370	0.561014	0.989200
H	4.485846	-1.286263	1.048441

Table S4. Optimized Cartesian coordinates in Å and ground-state SCF energies, E_{SCF}, in atomic units (a.u.) for the S_1/S_0 minimum-energy crossing point (MECP) geometry of $[\mathrm{BP4{-}H}]^-$ at the *ω*B97X-D/ma-*def*2-SV(P) level.

E_{SCF} = -1388.47789110			
C	0.098901	-1.703817	-0.565039
C	-1.249833	-1.554745	-1.029099
C	-2.109776	-0.566300	-0.580780
C	-1.659739	0.396392	0.390894
C	-0.359016	0.286148	0.858399
C	0.510869	-0.716302	0.415234
C	1.884679	-0.824794	0.884651
C	3.008411	-0.159316	0.281227
C	2.793649	0.685637	-0.826691
C	3.868155	1.330639	-1.429542
C	5.162399	1.144602	-0.940788
C	5.385528	0.306863	0.159411
C	4.321868	-0.342700	0.767981
O	2.139570	-1.588874	1.926902
O	0.886961	-2.608407	-0.938601
H	1.310192	-2.008128	2.199135
O	-3.319152	-0.575875	-1.178898
C	-4.527231	-0.289773	-0.491325
H	-4.737852	0.787871	-0.501176
H	-4.481177	-0.627495	0.553941
H	-5.312333	-0.840128	-1.030006
S	-2.606833	1.805020	1.052783
O	-1.584311	2.626500	1.735867
O	-3.594272	1.201615	1.977215
O	-3.206772	2.449690	-0.135337
H	-1.618144	-2.267074	-1.769837
H	-0.024901	1.047551	1.569068
H	1.776496	0.824392	-1.199201
H	3.692840	1.984267	-2.287068
H	6.004449	1.653683	-1.417350
H	6.400235	0.161722	0.538828
H	4.488319	-1.001980	1.621976

Table S5. Optimized Cartesian coordinates in Å and ground-state SCF energies, E_{SCF}, in atomic units (a.u.) for the S_3/S_2 minimum-energy crossing point (MECP) geometry of $[BP4–H]^-$ at the ωB97X-D/ma-*def*2-SV(P) level.

E_{SCF} = -1388.55672076			
C	-0.216422	-2.390303	-0.111570
C	-1.593661	-2.324942	-0.086102
C	-2.324128	-1.079635	0.005214
C	-1.582372	0.182503	0.091030
C	-0.213951	0.082074	0.078711
C	0.547004	-1.174665	0.000344
C	2.040585	-1.320049	0.028151
C	2.928073	-0.164084	-0.000516
C	2.697411	1.066569	-0.679471
C	3.667929	2.064910	-0.706511
C	4.895165	1.883028	-0.059912
C	5.150639	0.666463	0.601377
C	4.198891	-0.337591	0.615215
O	2.516073	-2.481575	0.064032
O	0.381178	-3.574135	-0.168873
H	1.371607	-3.345732	-0.065739
O	-3.616329	-1.359282	-0.029218
C	-4.726082	-0.510661	0.189307
H	-4.832668	0.191994	-0.657868
H	-4.573297	0.092385	1.100123
H	-5.587699	-1.184181	0.274385
S	-2.235971	1.888404	0.147822
O	-1.055768	2.753766	0.040630
O	-2.948256	1.998819	1.440878
O	-3.136039	1.951477	-1.031455
H	-2.174969	-3.246544	-0.134323
H	0.335971	1.021220	0.169809
H	1.758745	1.236792	-1.211973
H	3.461677	2.999647	-1.236418
H	5.648439	2.676565	-0.069982
H	6.108634	0.512083	1.108302
H	4.396065	-1.296180	1.101702

Table S6. Optimized Cartesian coordinates in Å and ground-state SCF energies, E_{SCF}, in atomic units (a.u.) for the S_2/S_1 minimum-energy crossing point (MECP) geometry of $[BP4–H]^{-}$ at the ωB97X-D/ma-*def*2-SV(P) level.

E_{SCF} = -1388.56833379			
C	-0.219164	-2.412488	-0.131418
C	-1.592658	-2.343300	-0.181351
C	-2.293985	-1.123884	-0.059205
C	-1.545477	0.098324	0.126022
C	-0.153326	0.012608	0.195625
C	0.554534	-1.195808	0.028546
C	2.020393	-1.333623	0.036033
C	2.926768	-0.169089	-0.005386
C	2.677223	0.980167	-0.786303
C	3.605108	2.017422	-0.825789
C	4.799015	1.932346	-0.102671
C	5.062461	0.787860	0.659287
C	4.144334	-0.255334	0.704828
O	2.508344	-2.506877	0.163209
O	0.359011	-3.620672	-0.265178
H	1.328799	-3.543557	-0.200660
O	-3.615193	-1.300650	-0.141628
C	-4.616089	-0.351422	0.186665
H	-4.648630	0.452745	-0.562850
H	-4.421592	0.104500	1.168498
H	-5.554818	-0.920160	0.196469
S	-2.213083	1.796418	0.223094
O	-1.018025	2.657553	0.223690
O	-2.979747	1.831915	1.489847
O	-3.047373	1.922783	-0.992988
H	-2.170142	-3.259513	-0.308048
H	0.382846	0.944843	0.378172
H	1.754132	1.062901	-1.364353
H	3.388708	2.903789	-1.428155
H	5.520659	2.753436	-0.132703
H	5.994848	0.710926	1.226667
H	4.348593	-1.155017	1.289134

S8. Further computational results

Table S7. Summary of vertical excitation energies, Δ*E*, oscillator strengths, *f*, and transition characters evaluated at the S_0 minimum-energy geometry. Tabulated values are at the *ω*B97X-D/ma-*def*2-SV(P) level; values in parentheses are at the ADC(2)/MP2/ma-*def*2-SV(P) level.

**Transition**	**Transition**	**Δ*E* (eV)**	***f***
$T_1 \leftarrow S_0$	*ππ**	3.183	0.000
$T_2 \leftarrow S_0$	*ππ**	3.745	0.000
$T_3 \leftarrow S_0$	*nπ**	3.872	0.000
$T_4 \leftarrow S_0$	*nπ**	4.139	0.000
$S_1 \leftarrow S_0$	*ππ**	4.272 (3.533)	0.256 (0.156)
$S_2 \leftarrow S_0$	*nπ**	4.357 (3.701)	0.010 (0.004)
$S_3 \leftarrow S_0$	*ππ**	4.756 (4.120)	0.365 (0.273)

We note that the lowest energy π/σ* and n/σ* states appear >8 eV at the Franck-Condon point.

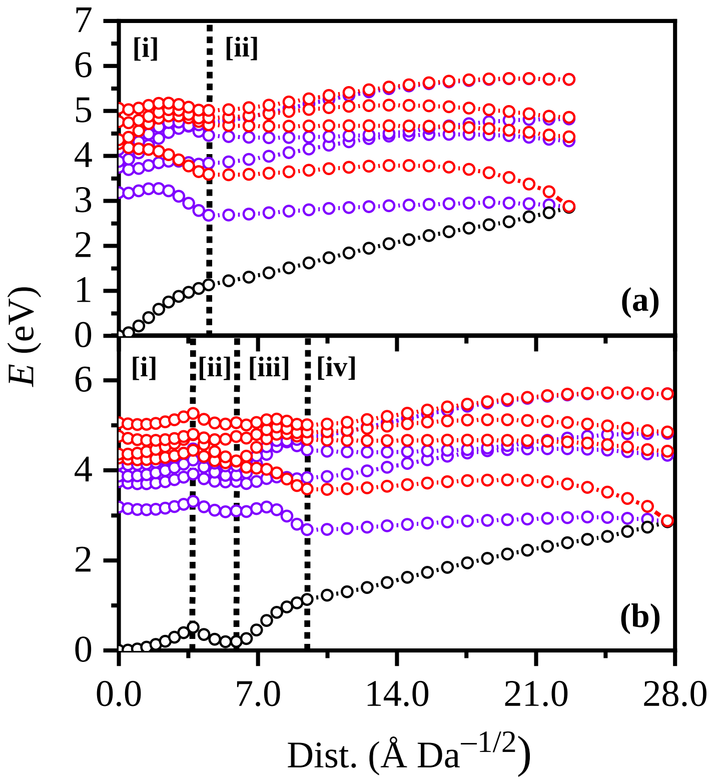

Figure S6. (a) Energies of the S_0 state (black) and excited singlet (red) and triplet (violet) states between **(i)** the S_0 and S_1 minimum-energy geometries, and **(ii)** the S_1 minimum-energy geometry and the S_1/S_0 MECP. **(b)** Energies of the S_0 state (black) and excited singlet states (red) between **(i)** the S_0 minimum-energy geometry and the S_3/S_2 MECP, **(ii)** the S_3/S_2 MECP and the S_2/S_1 MECP, **(iii)** the S_2/S_1 MECP and the S_1 minimum-energy geometry, and **(iv)** the S_1 minimum-energy geometry and the S_1/S_0 MECP. Points were generated *via* linear interpolation of internal coordinates (LIIC). Energies were evaluated at the ωB97X-D/ma-*def*2-SV(P) level.

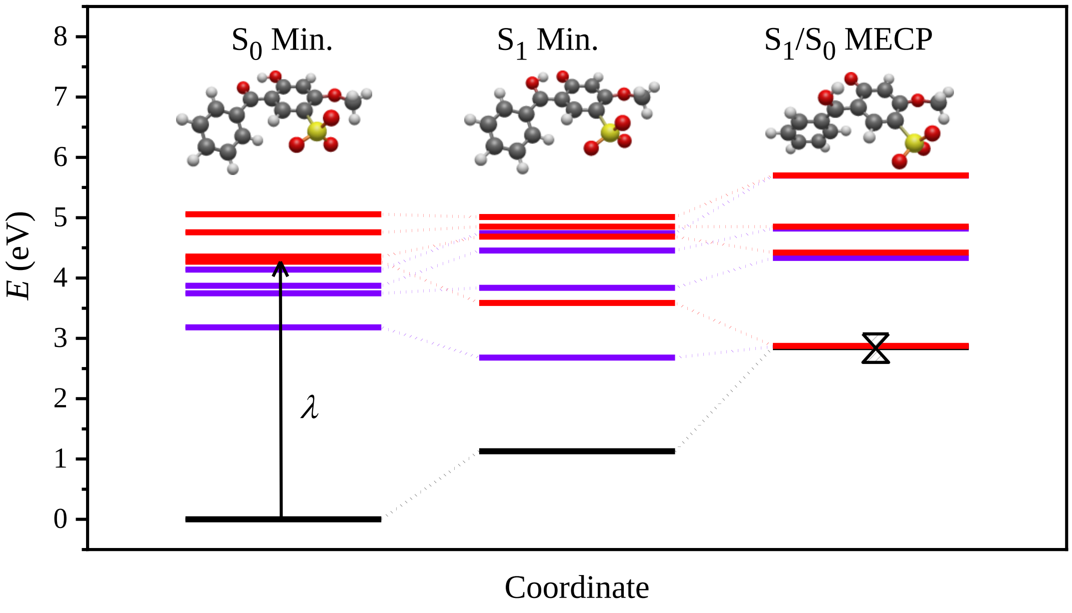

Figure S7. Energies of the S_0 state (black) and excited singlet (red) and triplet (violet) states at key geometries for the S_1 excitation scheme. MECP are denoted *via* a diabolo. Energies were evaluated at the *ω*B97X-D/ma-*def*2-SV(P) level.

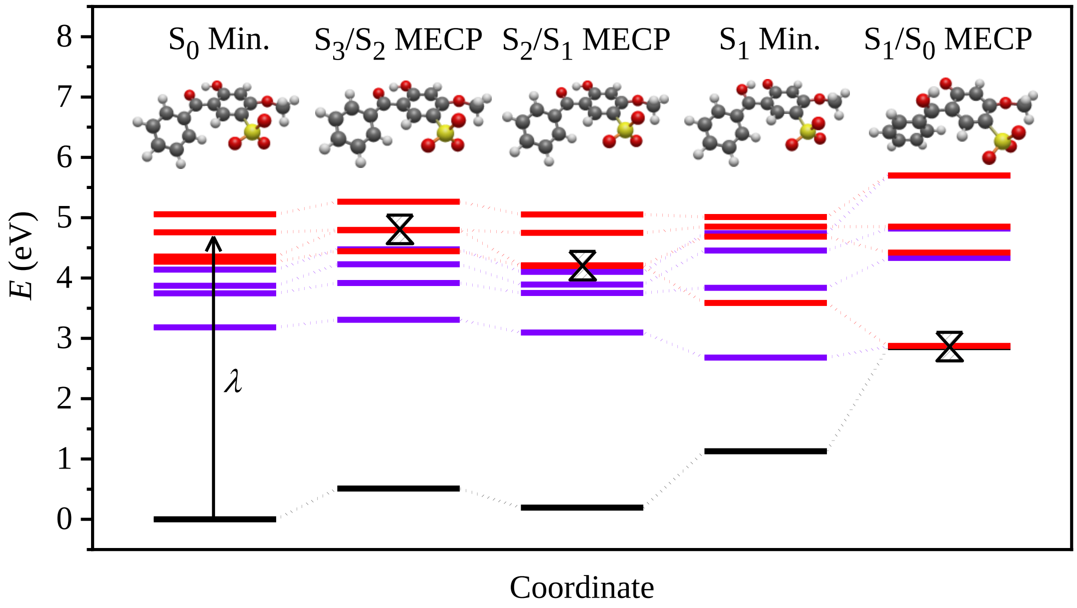

Figure S8. Energies of the S_0 state (black) and excited singlet (red) and triplet (violet) states at key geometries for the S_3 excitation scheme. MECP are denoted *via* a diabolo. Energies were evaluated at the ωB97X-D/ma-*def*2-SV(P) level.

S9. Schematic structure of deprotonated benzophenone-4

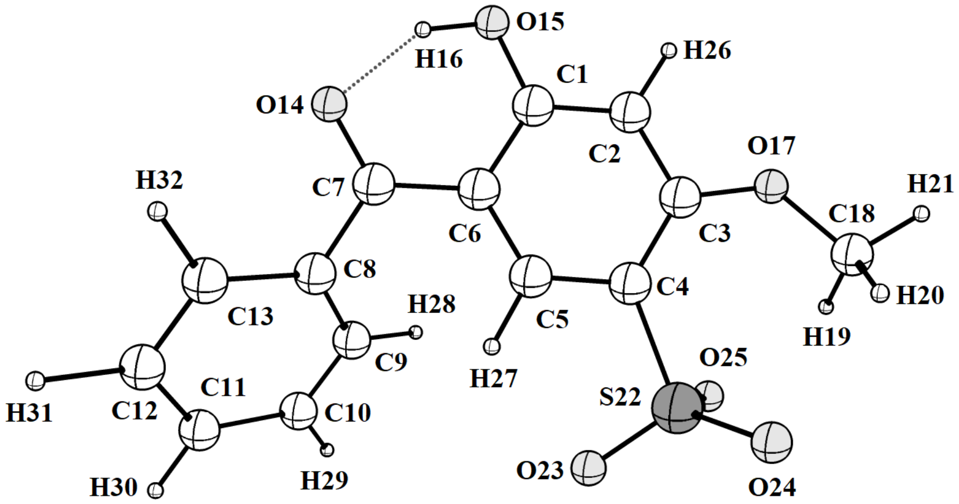

Scheme S1. Structure of deprotonated benzophenone-4 ($[BP4–H]^{–}$). All atoms are labelled.

References

(1) Wong, N. G. K.; Berenbeim, J. A.; Hawkridge, M.; Matthews, E.; Dessent, C. E. H. Mapping the Intrinsic Absorption Properties and Photodegradation Pathways of the Protonated and Deprotonated Forms of the Sunscreen Oxybenzone. *Phys. Chem. Chem. Phys.* **2019**, *21*, 14311–14321.

(2) Wong, N. G. K.; Berenbeim, J. A.; Dessent, C. E. H. Direct Observation of Photochemical Free Radical Production from the Sunscreen 2-Phenylbenzimidazole-5-Sulfonic Acid via Laser-Interfaced Mass Spectrometry. *ChemPhotoChem* **2019**, *3*, 1231–1237.

(3) Matthews, E.; Sen, A.; Yoshikawa, N.; Bergström, E.; Dessent, C. E. H. UV Laser Photoactivation of Hexachloroplatinate Bound to Individual Nucleobases: In Vacuo as Molecular Level Probes of a Model Photopharmaceutical. *Phys. Chem. Chem. Phys.* **2016**, *18*, 15143–15152.

(4) Sen, A.; Luxford, T. F. M.; Yoshikawa, N.; Dessent, C. E. H. Solvent Evaporation versus Proton Transfer in Nucleobase–Pt(CN)4,62− Dianion Clusters: A Collisional Excitation and Electronic Laser Photodissociation Spectroscopy Study. *Phys. Chem. Chem. Phys.* **2014**, *16*, 15490–15500.

(5) Antoine, R.; Dugourd, P. Visible and Ultraviolet Spectroscopy of Gas Phase Protein Ions. *Phys. Chem. Chem. Phys.* **2011**, *13*, 16494–16509.

(6) Sagoo, S. K.; Jockusch, R. A. The Fluorescence Properties of Cationic Rhodamine B in the Gas Phase. *J. Photochem. Photobiol. A Chem.* **2011**, *220*, 173–178.

(7) Cercola, R.; Matthews, E.; Dessent, C. E. H. Photoexcitation of Adenosine 5'-Triphosphate Anions in Vacuo: Probing the Influence of Charge State on the UV

Photophysics of Adenine. *J. Phys. Chem. B* **2017**, *121*, 5553–5561.

(8) Olsen, J. V; Macek, B.; Lange, O.; Makarov, A.; Horning, S.; Mann, M. Higher-Energy C-Trap Dissociation for Peptide Modification Analysis. *Nat. Methods* **2007**, *4*, 709–712.

(9) Neese, F. The ORCA Program System. *WIREs Comput. Mol. Sci.* **2012**, *2*, 73–78.

(10) Neese, F. Software Update: The ORCA Program System, Version 4.0. *WIREs Comput. Mol. Sci.* **2018**, *8*, e1327.

(11) Ahlrichs, R.; Bär, M.; Häser, M.; Horn, H.; Kölmel, C. Electronic Structure Calculations on Workstation Computers: The Program System Turbomole. *Chem. Phys. Lett.* **1989**, *162*, 165–169.

(12) Chai, J.-D.; Head-Gordon, M. Long-Range Corrected Hybrid Density Functionals with Damped Atom–Atom Dispersion Corrections. *Phys. Chem. Chem. Phys.* **2008**, *10*, 6615–6620.

(13) Hättig, C.; Weigend, F. CC2 Excitation Energy Calculations on Large Molecules Using the Resolution of the Identity Approximation. *J. Chem. Phys.* **2000**, *113*, 5154–5161.

(14) Hättig, C.; Köhn, A. Transition Moments and Excited-State First-Order Properties in the Coupled-Cluster Model CC2 Using the Resolution-of-the-Identity Approximation. *J. Chem. Phys.* **2002**, *117*, 6939–6951.

(15) Hättig, C. Geometry Optimizations with the Coupled-Cluster Model CC2 Using the Resolution-of-the-Identity Approximation. *J. Chem. Phys.* **2003**, *118*, 7751–7761.

(16) Köhn, A.; Hättig, C. Analytic Gradients for Excited States in the Coupled-Cluster Model CC2 Employing the Resolution-of-the-Identity Approximation. *J. Chem. Phys.* **2003**, *119*, 5021–5036.

(17) Levine, B. G.; Coe, J. D.; Martínez, T. J. Optimizing Conical Intersections without Derivative Coupling Vectors: Application to Multistate Multireference Second-Order Perturbation Theory (MS-CASPT2) †. *J. Phys. Chem. B* **2008**, *112*, 405–413.

(18) Zheng, J.; Xu, X.; Truhlar, D. G. Minimally Augmented Karlsruhe Basis Sets. *Theor. Chem. Acc.* **2011**, *128*, 295–305.

(19) Stoychev, G. L.; Auer, A. A.; Neese, F. Automatic Generation of Auxiliary Basis Sets. *J. Chem. Theory Comput.* **2017**, *13*, 554–562.

(20) Berenbeim, J. A.; Wong, N. G. K.; Cockett, M. C. R.; Berden, G.; Oomens, J.; Rijs, A. M.; Dessent, C. E. H. Unravelling the Keto–Enol Tautomer Dependent Photochemistry and Degradation Pathways of the Protonated UVA Filter Avobenzone. *J. Phys. Chem. A* **2020**, *124*, 2919–2930.

(21) Cercola, R.; Matthews, E.; Dessent, C. E. H. Near-Threshold Electron Transfer in Anion-Nucleobase Clusters: Does the Identity of the Anion Matter? *Mol. Phys.* **2019**, 1–10.

(22) Scheer, A. M.; Mukarakate, C.; Robichaud, D. J.; Ellison, G. B.; Nimlos, M. R. Radical Chemistry in the Thermal Decomposition of Anisole and Deuterated Anisoles: An Investigation of Aromatic Growth. *J. Phys. Chem. A* **2010**, *114*, 9043–9056.

(23) Zhang, X. Mass Spectrometric and Theoretical Studies on Dissociation of the CS Bond in the Benzenesulfonic Acid and Benzenesulfinic Acid Anion Series: Homolytic Cleavage vs Heterolytic Cleavage. *J. Mol. Struct.* **2012**, *1028*, 1–6.

(24) Ben-Ari, J.; Etinger, A.; Weisz, A.; Mandelbaum, A. Hydrogen-Shift Isomerism: Mass Spectrometry of Isomeric Benzenesulfonate and 2-, 3- and 4-Dehydrobenzenesulfonic Acid Anions in the Gas Phase. *J. Mass Spectrom.* **2005**, *40*, 1064–1071.

(25) Binkley, R. W.; Flechtner, T. W.; Tevesz, M. J. S.; Winnik, W.; Zhong, B. Rearrangement of Aromatic Sulfonate Anions in the Gas Phase. *Org. Mass Spectrom.* **1993**, *28*, 769–772.
